# LKB1 and AMP-activated protein kinase: regulators of cell polarity

**DOI:** 10.1111/j.1365-2443.2012.01629.x

**Published:** 2012-08-14

**Authors:** Atsushi Nakano, Seiji Takashima

**Affiliations:** Department of Molecular Cardiology, Osaka University Graduate School of MedicineSuita, Osaka, 565-0871, Japan

## Abstract

Adenosine monophosphate–activated protein kinase (AMPK), a metabolic protein kinase, and its upstream kinase LKB1 play crucial roles in the establishment and maintenance of cell polarity. Although the shapes of polarized cells display extraordinary diversity, the key molecules involved in cell polarity are relatively well conserved. Here, we review the mechanisms and factors responsible for organizing cell polarity and the role of LKB1 and AMPK in cell polarity.

## Introduction

There is growing evidence that AMP-activated protein kinase (AMPK) and its upstream kinase liver kinase B1 (LKB1) have important roles in cell polarization (Baas *et al*. 2004a; Brenman 2007; Jansen *et al*. 2009; Mirouse & Billaud 2011; Sebbagh *et al*. 2011), and cell polarity is implicated in the differentiation and proliferation of single cells and multicellular organs. Cell polarity is initiated when nonpolarized cells, including zygotes, immature cells, floating cells and cultured cells, react to extrinsic polarization cues such as sperm insertion or the development of the extracellular matrix (ECM). The result is the asymmetrical redistribution of cellular components and, occasionally, the reorganization of the cytoskeleton to change the shape of the cell, thus establishing cell polarity. Accordingly, early development is a good model to study the mechanism of the establishment of cell polarity, and *Caenorhabditis elegans* zygotes (Lyczak *et al*. 2002), *Drosophila* oocytes (Pellettieri & Seydoux 2002) and cultured cells (Li & Gundersen 2008) have been widely used to analyze the mechanisms required for establishing anterior–posterior polarity or front-rear polarity. However, mature cells in various organs also display cell polarity and can respond to extrinsic polarization cues such as growth factors; the microtubule cytoskeleton generally responds to these cues in the process of cell polarization. Epithelial cells are the archetypal cell type that displays apical–basal polarity. In vertebrates, the basolateral and basal surfaces of these cells have very different cell surface compositions from each other, and there are tight junctions at the apical-most side of the lateral surfaces, which tightly connect adjacent cells and limit fluid and molecules from permeating vertically. In addition, adherens junctions that are located immediately beneath the tight junctions and desmosomes serve as scaffolds for binding the actin cytoskeleton and intermediate filaments, respectively, whereas hemi-desmosomes at the basement membrane connect to the ECM via integrins (Bryant & Mostov 2008). These interactions via transmembrane structures between adjacent cells or between cells and the ECM play important roles in maintaining cell polarity (Iden & Collard 2008). Both AMPK and LKB1 are required for establishing and maintaining cell polarity in these various cell types. Here, we summarize the function of LKB1 and AMPK and their effects on the regulation of cell polarity.

## Molecular characteristics and physiological role of LKB1

The *LKB1* gene was cloned in 1997 using comparative genomic hybridization of polyp DNA from patients with Peutz–Jeghers syndrome (PJS) (Hemminki *et al*. 1997). Patients with PJS develop multiple hamartomatous polyps and also have a markedly increased risk of developing malignant tumors. One of the causes of PJS was shown as a loss-of-function mutation in the human *LKB1* gene (Hemminki *et al*. 1998), which is also known as serine–threonine protein kinase 11 (*STK11*) (Jenne *et al*. 1998), suggesting that LKB1 protein acts as a tumor suppressor.

**Figure 1:**
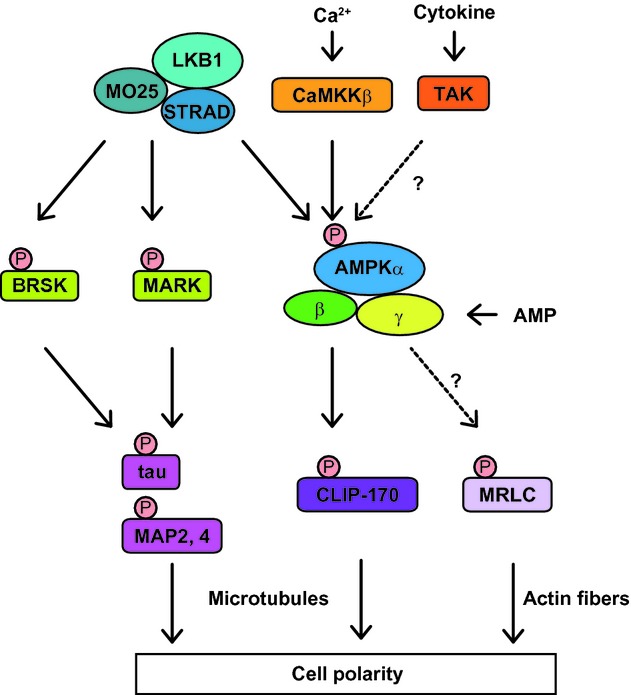
Adenosine monophosphate–activated protein kinase (AMPK) signaling pathways related to cell polarity. The complex of LKB1, STe20-Related ADaptor (STRAD) and MO25 directly phosphorylates and activates BRSK, microtubule affinity-regulating kinase (MARK) and AMPK, which are AMPK-related protein kinases. AMPK is activated by the direct binding of AMP to its γ subunit; thus, the activity of AMPK reflects the energy level of the cell. AMPK is also phosphorylated by CaMMKβ in response to increased intracellular Ca^2+^ concentrations. The phosphorylation of tau, MAP2 and MAP4 by activated BRSK and MARK and the phosphorylation of CLIP-170 by AMPK regulate cell polarity via microtubule dynamics. The dotted line with a question mark is a branch that requires further investigation. MAP, microtubule-associated proteins.

The *LKB1* gene is expressed in a variety of fetal and adult tissues, as determined by Northern blot analysis (Jenne *et al*. 1998). LKB1 has an N-terminal regulatory domain in the most N-terminal region after the kinase domain and a C-terminal regulatory domain in its most C-terminal region. LKB1 also has two nuclear localization signals (NLSs) in its N-terminal region, and LKB1 without an NLS remains in the cytoplasm, even though wild-type LKB1 is localized to both the cytoplasm and the nucleus (Sapkota *et al*. 2002). To be activated, LKB1 requires the adaptor proteins STe20-Related ADaptor (STRAD) and mouse protein 25 (MO25) (Fig. 1) (Hawley *et al*. 2003). Although the single expressions of LKB1, STRAD or MO25 show their localization in the nucleus, the complex of these three components results to localize to the cytoplasm (Baas *et al*. 2003; Boudeau *et al*. 2003). LKB1 is thought to be constitutively activated in cells, even under conditions in which AMPK is activated (Alessi *et al*. 2006), an idea derived from the finding that the LKB1 complex is not stimulated by AMP *in vitro* and that LKB1 activity is not variable in different cell lines (Woods *et al*. 2003; Lizcano *et al*. 2004; Shaw *et al*. 2004).

Genetic studies by several groups have suggested that several protein kinases in *Saccharomyces cerevisiae*, elongated morphology□1 (Elm1), Snf1-activating kinase□1 (Sak1, formerly known as Pak1) and target of Sbf3 (Tos3), were capable of phosphorylating the catalytic domain of Snf1, the homologue of mammalian AMPK (Hong *et al*. 2003; Nath *et al*. 2003; Sutherland *et al*. 2003). Although these three kinases do not have obvious mammalian homologues, they displayed moderate similarity to LKB1 and Ca^2+^/calmodulin-dependent protein kinase kinase β (CaMKKβ). Using biochemical assays, LKB1 (Hawley *et al*. 2003; Woods *et al*. 2003) and CaMKKβ (Hawley *et al*. 2005; Hurley *et al*. 2005; Woods *et al*. 2005) were shown to phosphorylate AMPKα at Thr 172 in the catalytic domain. Thus, AMPK phosphorylation by LKB1 is a conserved substrate-kinase reaction from yeast to mammals.

A kinome analysis using public and proprietary genomic, complementary DNA and expressed sequence tags showed 14 AMPK-related protein kinases (AMPKα1, AMPKα2, BRSK1, BRSK2, NUAK1, NUAK2, QIK, QSK, SIK, MARK1, MARK2, MARK3, MARK4 and MELK) that contain a homologous catalytic domain (Manning *et al*. 2002). Indeed, the LKB1 complex phosphorylated the conserved threonine in the catalytic domain of each of the 14 AMPK-related protein kinases *in vitro* (Lizcano *et al*. 2004). These findings suggest that LKB1 is a master kinase of these 14 AMPK-related protein kinases and that these kinases also may mediate some of the physiological functions of LKB1.

## Molecular characteristics and physiological role of AMPK

Adenosine monophosphate–activated protein kinase is a serine–threonine protein kinase that acts as a sensor to gauge the intracellular energy status of eukaryotic cells. AMPK was initially discovered as a kinase that phosphorylates and inactivates both acetyl-CoA carboxylase and HMG-CoA reductase, which are related to lipid biosynthesis pathways, resulting in the production of energy (Carling *et al*. 1987). AMPK has been reported to phosphorylate many substrates associated with glucose and lipid metabolism, transcription, cell growth and cell polarity (Mihaylova & Shaw 2011).

Living cells store energy as ATP and ADP and release a phosphate group by hydrolyzing ATP to ADP or ADP to AMP. Therefore, the levels of AMP and ADP increase when cells are stressed, and the cells must accurately sense the levels of them to respond appropriately to this stress. Although it is difficult to determine the concentration of free AMP in living cells, an estimated concentration can be calculated by measuring the levels of ATP, phosphocreatine and creatine. It has been reported that the calculated concentration of AMP in human skeletal muscle is much lower than that of ATP (10^4^-fold) and ADP (10^2^-fold) and changes (25- to 45-fold up) more dramatically than that of ATP (<20% down) and ADP (4- to 6-fold up) after exercise, making AMP the best indicator of cellular stress (Hardie *et al*. 2011). AMPK has a high sensitivity to these adenine nucleotide changes through its direct binding to ATP, ADP and AMP. AMPK is activated by various extracellular stresses that either reduce the catabolic generation of ATP (e.g., ischemia, glucose depletion or oxidative stress) or increase the metabolic consumption of ATP (e.g., muscle contraction) (Hardie 2007).

Adenosine monophosphate–activated protein kinase is a heterotrimer comprising a catalytic subunit (α) and two regulatory subunits (β and γ). Once cells are exposed to conditions that are stressful or harmful, AMPK changes its conformation through the direct binding of an adenine nucleotide to the γ regulatory subunit and/or is activated through the phosphorylation of Thr172 of the catalytic α subunit by an upstream protein kinase (i.e., LKB1 or CaMKKβ) (Fig. 1). However, there is still little evidence of how these extracellular stresses lead to the activation of the upstream kinase of AMPK.

## LKB1 and AMPK regulate cell polarity in *Caenorhabditis elegans*

**Figure 2:**
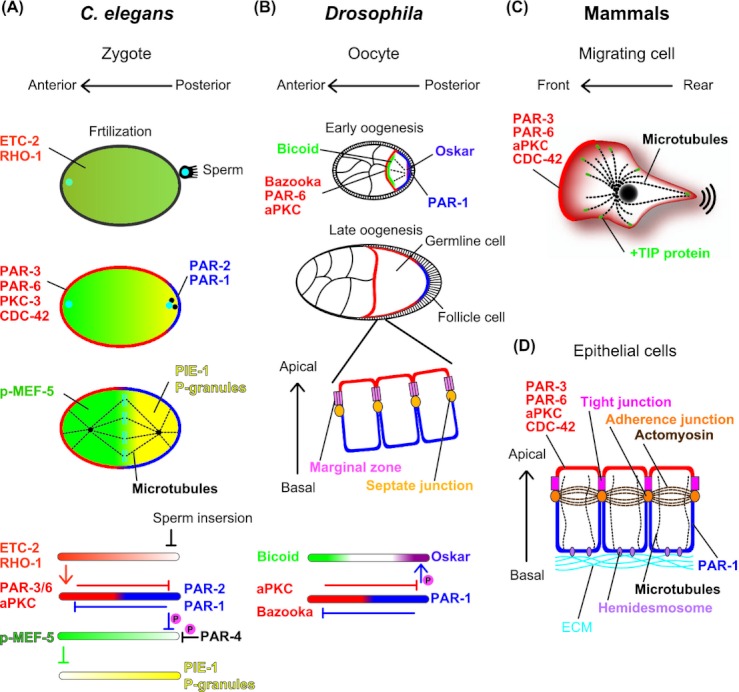
Anterior–posterior and apical–basal polarity in various cells. (A) The expression pattern of PAR and its related proteins during the establishment of anterior–posterior cell polarity in the *Caenorhabditis elegans* zygote after fertilization. MEF-5 (green) and PIE-1 (yellow) are distributed uniformly in the cytosol before fertilization. The anterior region is defined as the side opposite from sperm insertion, which triggers the exclusion of both ETC-2 and RHO-1 (orange) from the posterior cytoplasm. ETC-2 and RHO-1 activate the complex PAR-3/PAR-6/PKC-3/CDC-42 (red) and mediate its localization to the anterior cortex, and the complex PAR-3/PAR-6/PKC-3/CDC-42 excludes both PAR-1 and PAR-2 (blue) from the anterior cortex. PAR-1 and PAR-4 phosphorylate MEF-5, and phosphorylated MEF-5 localizes to the anterior cytoplasm. Phosphorylated MEF-5 also excludes PIE-1 and P granules from the anterior cytoplasm. Blue dots, pronuclei; black dots, microtubule-organizing centers; black dotted lines, microtubules. (B) The expression pattern of PAR and associated proteins in the *Drosophila* oocyte. The patterning of the anterior–posterior axis in *Drosophila* develops during oocytogenesis before fertilization. The mRNAs of the maternal-effect gene O*skar* (purple) and Bicoid (green) are localized to posterior and anterior regions of the germ-line cell by the transportation of microtubules, respectively. After the activation of the maternal-effect genes, PAR-1 (blue) and the Bazooka/PAR-6/aPKC complex (red) localize to the posterior and anterior of the germ-line cell, respectively. PAR-1 stabilizes the Oskar protein directly by phosphorylation; aPKC expression at the anterior excludes PAR-1 from the anterior cortex, and PAR-1 excludes Bazooka from the posterior cortex. In the follicle cells arranged around the germ-line cell, aPKC expression on the apical side also excludes PAR-1, and PAR-1 excludes Bazooka from the basal side. Bazooka stabilizes the septate junction (yellow). (C) Polarized mammalian migrating cell expressing PAR and its related proteins at the leading edge, representing front-rear polarity. Black dotted lines, microtubules; green dots, microtubule plus-end proteins. (D) Apical–basal polarization observed in mammalian epithelial cells. The PAR-3/PAR-6/aPKC/CDC-42 complex (red) localizes to the apical surfaces and PAR-1 (blue) to the basolateral and basal surfaces. The lateral membranes contain tight junctions (pink) and adherens junctions (ocher), anchoring actomyosin (brown dotted lines). The basal surfaces bind to the extracellular matrix (ECM) (light blue solid line) via hemidesmosomes (light pink).

In *Caenorhabditis elegans*, investigations of cell polarity have been focused on the anterior–posterior polarity defined by the asymmetric division of the one-cell zygote. Embryos exhibit their anterior–posterior polarity shortly after fertilization (Fig. 2). The delivery point of the fertilizing sperm, named the posterior site, initially excludes ETC-2, a potential guanine nucleotide-exchange factor for RHO-1, from the posterior cortex, which triggers the local activation of RHO-1, one of the Rho GTPases, at the anterior cortex (Motegi & Sugimoto 2006). The cues from the sperm-donated centrosome may inhibit and break the actomyosin meshwork, leading to the formation of anterior–posterior polarity (Munro *et al*. 2004); however, the precise mechanism is unknown. The role of LKB1 in cell polarity was first reported in a genetic screen for mutations affecting the timing and patterns of cleavage in early *C. elegans* embryos (Kemphues *et al*. 1988). The authors reported a characteristic phenotype in the early development of *C. elegans*: the embryos showed abnormally equal and synchronous cell divisions, suggesting that the abnormal partitioning of the cytosol had occurred. Kemphues *et al*. identified their responsible genes named *partitioning defective* (*par*). Loss-of-function mutations of the *par* genes abolish the tightly controlled polar distribution of maternally expressed regulatory proteins, resulting in severe defects in cell fate specification (Schneider & Bowerman 2003). *Par-1* encodes a serine–threonine kinase and has sequence identity with microtubule affinity-regulating kinase (MARK), which is a member of the AMPK subfamily and phosphorylates microtubule-associated proteins (MAP). *Par-2* encodes a protein with a RING finger domain that may act in the ubiquitination pathway. Both *Par-3* and *Par-6* encode proteins with PDZ domains, suggesting that they act as scaffold proteins. *Par-4*, which is a homologue of LKB1 in *C. elegans*, encodes a serine–threonine protein kinase. *Par-5* encodes a member of the 14-3-3 family of proteins (Table 1). Each PAR protein distributes characteristically in the asymmetrically dividing cells of the early germ-line lineage of *C. elegans* and plays a crucial role in anterior–posterior cell polarity. PAR-1 localizes to the posterior cortex by associating with PAR-2 (Boyd *et al*. 1996), PAR-3 and PAR-6 localize to the anterior cortex with PKC-3 (atypical protein kinase C), and PAR-4 and PAR-5 remain symmetrically localized to the cortex and cytosol (Goldstein & Macara 2007). The loss-of-function PAR alleles have suggested a complicated hierarchy; however, all of the PAR proteins are necessary for proper PAR-1 localization, suggesting that PAR-1 is the most downstream effector of the other PAR proteins (Fig. 2) (Baas *et al*. 2004b).

**Table 1:** Polarity-related proteins in *Caenorhabditis elegans*, *Drosophila melanogaster* and mammals

*C. elegans*	*D. melanogaster*	Mammals
PAR-1	PAR-1	MARK3/PAR-1a
		MARK2/PAR-1b
		MARK1/PAR-1c
		MARK4
PAR-2	Not identified	Not identified
PAR-3	Bazooka	ASIP
PAR-4	dLKB1	LKB1/STK11
PAR-5	14-3-3ε	14-3-3β
PAR-6	PAR-6	PAR-6α
		PAR-6β
		PAR-6γ
PKC-3	aPKC	aPKCλ
		aPKCζ
CDC-42	CDC-42	CDC-42
aak-1	AMPKα	AMPKα1
	aak-2	AMPKα2

ASIP, Atypical PKC isotype-specific interacting protein; MARK, microtubule affinity-regulating kinase.

*Par-4* (homologue of LKB1) is required for cytoplasmic division during the early stages of development. PAR-4 is cortically distributed in the cytoplasm from the 1-cell stage and is present in smaller amounts at later stages in *C. elegans* (Watts *et al*. 2000). Maternal-effect lethal mutations in PAR-4 that result in the failure of intestinal cells to differentiate have been shown to affect several aspects of cell polarity (Morton *et al*. 1992). A life-span analysis for mutated *par-4* and the AMP-activated kinase α2 catalytic subunit (*aak-2*), the homologue of AMPK in *C. elegans*, indicated that *par-4* did not affect the life span. However, *aak-2* mutations shorten the life span by causing the rapid consumption of stored energy in *C. elegans* (Narbonne & Roy 2009).

There are molecular gradients in the single-cell cytoplasm in which MEX-5 (muscle excess 5) is dominant in the area destined to become the somatic blastomere, whereas PIE-1 (pharynx and intestine in excess protein 1) is dominant in the opposite area, which is destined to become the germ-line blastomere. MEX-5 is an RNA-binding protein that is inherited by the somatic blastomere and segregates the P granules and PIE-1 in response to PAR-1 asymmetry. PAR-1 and PAR-4 phosphorylate MEX-5 and cause the rapid movement of MEX-5 in the cytoplasm, resulting in cytoplasmic asymmetry of MEX-5 (Tenlen *et al*. 2008; Griffin *et al*. 2011). P granules are cytoplasmic RNA-rich granules that are mostly localized to the germ-line, and the asymmetric segregation of the P granules during early division in *C. elegans* is a classic example of cytoplasmic patterning and cell polarity. However, the physiological role of the P granules is not fully understood because a recent investigation using P granule mutants showed that the P granules are not required to specify the germ-line (Gallo *et al*. 2010).

Although *C. elegans* is a fundamental model for the analysis of anterior–posterior cell polarity, the precise mechanisms of establishing cell polarity are still not fully understood. However, it is indisputable that the PAR proteins are crucial regulators of cell polarity and that PAR-1 and PAR-4 play important roles in this process. Important findings about the par proteins that clarify the mechanisms of cell polarity have been accumulating and will provide a better understanding of cell polarity.

## LKB1 and AMPK regulate cell polarity in *Drosophila*

In contrast to *C. elegans*, the *Drosophila* oocyte exhibits anterior–posterior polarity from the early developing oogenesis stage and also apical–basal polarity in the late oogenesis stage and embryonic stage. PAR proteins homologous to those in *C. elegans*, except for PAR-2, have also been identified in *Drosophila*, and they regulate anterior–posterior cell polarity in the oocyte (Table 1).

The *Drosophila par-1* gene is expressed early in the both germ-line cells and somatic follicle cells in the ovary and accumulates at the anterior cortex in stage 9 oocytes (Fig. 2). The deletion of *Drosophila par-1* appears to be zygotic lethal, and germ-line clones result in the arrest of oocytes before axis formation. Although *Drosophila* PAR-1 seems to affect microtubule organization the most directly, there is no evidence that *C. elegans* PAR-1 affects microtubule organization, and *C. elegans* polarity is resistant to microtubule-inhibiting drugs (Kemphues 2000).

*Dlkb1*, a homologue for *PAR-4* in *Drosophila melanogaster*, was also identified as being required for both early anterior–posterior polarity and apical–basal polarity (Martin & St Johnston 2003). These researchers showed that immunoprecipitated *Drosophila* PAR-1 phosphorylated recombinant LKB1 *in vitro*, even though PAR-1/MARK is phosphorylated by LKB1 in mammalian cells. Thus, the hierarchy of PAR proteins for establishing cell polarity in *Drosophila* might be different from that in mammals. The mutation of *dlkb1* resulted in disrupted spindle formation and asymmetric division in larval neuroblasts (Bonaccorsi *et al*. 2007). PAR-4 is essential for retinal cell apical–basal polarity during remodeling in eye formation; however, AMPK might not be a direct target of PAR-4 in the retina (Amin *et al*. 2009). Additionally, many investigators have reported that LKB1 is essential for cell polarity in nonmammalian cells (Tenlen *et al*. 2008; Kim *et al*. 2010).

The PAR–aPKC complex is also conserved in *Drosophila*; Bazooka (PAR3), PAR-6 and aPKC (PKC-3) localize to the anterior cortices of oocytes (Vaccari & Ephrussi 2002; Benton & St Johnston 2003) and to the apical and apicolateral membranes of follicle epithelia (Kim *et al*. 2009).

Abnormal cell polarity and mitosis were observed in the epithelia of both *AMPK* and *LKB1* null mutant *Drosophila*, and a phosphomimetic mutant of myosin regulatory light chain (MRLC) rescued the phenotype (Lee *et al*. 2007). These findings suggest that the actin–myosin cytoskeleton complex might be regulated in some circumstances in which AMPK is activated; although the authors showed that AMPK directly phosphorylates the MRLC, the phosphomimetic transgene did not rescue all of the phenotypes. Furthermore, in a recent study using mammalian pancreatic cells, the inhibition of LKB1 did not affect the MRLC phosphorylation status (Hezel *et al*. 2008), and the inhibition of AMPK in vascular smooth muscle cells increased MRLC phosphorylation (Horman *et al*. 2008). These findings imply that the MRLC may not be directly phosphorylated by AMPK (Bultot *et al*. 2009) but instead mediate the polarity signal from AMPK to the actin cytoskeleton (Mirouse & Billaud 2011).

## LKB1 and AMPK regulate cell polarity in mammals

Because of the difficulties of working with fertilized mammalian eggs, as opposed to *C. elegans* zygotes or *Drosophila* oocytes, most investigators have examined the mechanisms of the establishment and maintenance of cell polarity in mammals using cultured epithelial cells. Cultured cells displaying directional cell migration facilitate the identification of the mechanism of front-rear polarity, and epithelial cells as Madin-Darby canine kidney (MDCK) cells are good models for understanding apical–basal polarity (Fig. 2).

An elegant investigation showed the physiological function of LKB1 in single-cultured cells. Intestinal epithelial cancer cell lines that remained unpolarized owing to a lack of STRAD yet could be polarized by inducible STRAD were constructed. Upon STRAD induction, the unpolarized cells rapidly polarized, remodeling their actin cytoskeleton to form an apical brush border when LKB1 was activated by the induced STRAD (Baas *et al*. 2004a). These findings suggest that LKB1 is a major regulator of cell polarity. MDCK epithelial cells require Ca^2+^ for the assembly of both tight junctions and adherens junctions, which can be manipulated by changing the concentration of calcium in the medium. Two studies using these calcium-switch experiments showed that AMPK is essential for tight junction assembly; however, these pathways might require the calcium–CaMKKβ axis independently of LKB1 (Zhang *et al*. 2006; Zheng & Cantley 2007).

The MARK family of proteins, homologues of PAR-1 in mammals, are also involved in the maintenance of cell polarity in epithelial cells (Bohm *et al*. 1997) and neural cells (Sapir *et al*. 2008). MARKs are reported to phosphorylate MAP 2 and 4 and tau, increasing the dynamic instability of microtubules (Ebneth *et al*. 1999). MARKs have a catalytic domain near its N terminus that is followed by an autoregulatory domain and a kinase-associated domain. MARKs are known to be members of the AMPK subgroup of the Ca^2+^/calmodulin-dependent protein kinase (CAMK) family and to promote the detachment of phosphorylated MAPs from microtubules, resulting in the regulation of microtubule dynamics and microtubule-based intracellular transport. Tau protein is one of the most famous substrates for MARK, and hyperphosphorylated Tau aggregates in neural cells, causing defects in neural transport and forming filamentous structures that consist of neurofibrillary tangles, which are the hallmark of Alzheimer's disease (Ballard *et al*. 2011). However, the precise effects of MARKs on microtubule dynamics and the mechanisms that underlie these effects are not fully understood (Hayashi *et al*. 2012).

Atypical PKC isotype-specific interacting protein (ASIP), a homologue of PAR-3 in mammals, and PAR-6 are also highly conserved from *C. elegans* to humans (Table 1) and localize at the tight junction near the apical side of mammalian epithelial cells, forming a complex with aPKC and cell division control protein 42 (CDC-42), one of the small GTPases. The complex is essential for maintaining apical–basal polarity (Izumi *et al*. 1998; Joberty *et al*. 2000).

Of the 14 AMPK-related protein kinases, BRSK, MARK1, MARK2, MARK3, MARK4 and AMPK play roles in regulating cell polarity (Matenia & Mandelkow 2009; Shackelford & Shaw 2009); however, there are few reported relationships between other kinases and cell polarity. The catalytic domains of these 14 AMPK-related protein kinases are phosphorylated by upstream kinases, and three AMPK kinases have been identified to date, namely LKB1, CaMKKβ and transforming growth factor-β-activated kinase 1 (TAK1) (Bright *et al*. 2009). CaMKKβ has been identified as an AMPK kinase (Hawley *et al*. 2005; Hurley *et al*. 2005; Woods *et al*. 2005), phosphorylating and activating AMPK in response to increased intracellular Ca^2+^ concentrations. TAK1 was very recently implicated in the regulation of AMPK activity in cells and in the heart, although the physiological conditions under which TAK1 regulates AMPK are unclear (Momcilovic *et al*. 2006; Xie *et al*. 2006) (Fig. 1).

## AMPK regulates microtubule dynamics by phosphorylation of CLIP-170

**Figure 3:**
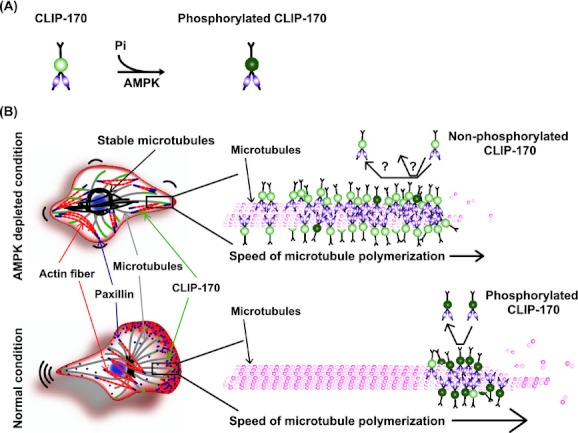
Adenosine monophosphate–activated protein kinase (AMPK) phosphorylates CLIP-170 and establishes cell polarity. (A) AMPK phosphorylates CLIP-170 at Ser 311 of its coiled-coil region. (B) The phosphorylation of CLIP-170 by AMPK is required for the efficient polymerization of microtubules and the establishment of cell polarity (lower panel). Phosphorylated CLIP-170 rapidly detaches from the microtubule lattice, contributing to efficient microtubule polymerization. In contrast, the inhibition of AMPK results in the prolonged and enhanced accumulation of nonphosphorylated CLIP-170 on the microtubule lattice, leading to the disturbance of microtubule polymerization and cell polarity (upper panel).

Although there is growing evidence that LKB1 and AMPK have pivotal roles in the establishment of cell polarity, as described above, the direct downstream targets of AMPK associated with cell polarity remain unclear. Using two-step column chromatography combined with an *in vitro* kinase reaction, cytoplasmic linker protein of 170 Da (CLIP-170) was recently identified as a novel substrate for AMPK in mammalian cells and was shown to play a key role in the cell polarity mediated by AMPK (Nakano *et al*. 2010). CLIP-170 was originally identified as a protein that mediates the binding of endocytic vesicles to microtubules in HeLa cells (Rickard & Kreis 1990; Pierre *et al*. 1992). CLIP-170 also acts as one of the microtubule plus-end tracking proteins (Perez *et al*. 1999; Schuyler & Pellman 2001). AMPK was shown to phosphorylate CLIP-170 directly at Ser 311, and phosphorylated CLIP-170 localizes closer to the distal end of microtubules than nonphosphorylated CLIP-170. In contrast, decreasing the level of phosphorylated CLIP-170 using AMPK inhibitors or depleting AMPK with siRNA shifted the dissociation pattern of CLIP-170 at the distal end of the microtubules, resulting in a disturbance of cell polarity (Nakano *et al*. 2010) (Fig. 3). Another report clearly showed that CLIP-170 that is not phosphorylated in its serine-rich region, including Ser 311, changes its conformation and increases its binding affinity to microtubules. These reports suggest that the phosphorylation of CLIP-170 by AMPK may decrease its affinity for tubulins or shift the binding site to more distal ends of the microtubules than the binding site of nonphosphorylated CLIP-170; however, more detailed reports are necessary to clarify the precise mechanisms (Lee *et al*. 2010).

CLIP-190, an ortholog of CLIP-170 in *Drosophila*, is reported to localize to the distal end of growing microtubules, similar to CLIP-170, in cultured cells (Dzhindzhev *et al*. 2005). A recent study showed that CLIP-190 is localized to the posterior of the oocyte in a manner that is dependent on the Oskar protein and the actin cytoskeleton (Sanghavi *et al*. 2012). However, the role of CLIP-190 in cell polarity has not been fully investigated.

## AMPK controls cell polarity and cell migration

Controlling microtubule dynamics by the phosphorylation of CLIP-170 via AMPK affects the stability of microtubules (Nakano *et al*. 2010). Microtubules function as a railway for the efficient transport of a large amount of cargo between the cytoplasm and the cell cortex. Thus, increasing the stability of microtubules may lead to a disturbance of the transportation of various substances within cells. It has been reported that the inhibition of AMPK activity disturbs anterior–posterior polarity, particularly front-rear polarity, and that a phosphomimetic mutant of CLIP-170 successfully rescues these phenomena, suggesting that the AMPK–CLIP-170 axis is important for establishing cell polarity (Fig. 3).

A recent investigation also showed that AMPK is required for establishing the growth of single neurites and their differentiation into axons (Amato *et al*. 2011). In this study, AMPK was shown to phosphorylate kinesin light chain (KLC) 2 of kinesin 1 at both Ser 539 and Ser 575, disrupting the association between the phosphatidylinositol 3-kinase regulatory subunit p85 and KLC2 and resulting in the suppression of axon initiation and neuronal polarization. Therefore, AMPK may establish and control cell polarity via the microtubule cytoskeleton.

## Conclusions and future directions

In this review, we have presented the recent advances in knowledge about the function of the LKB1-AMPK pathway in the establishment of anterior–posterior cell polarity. Over the 15 years since the discovery of *LKB1* as one of the genes responsible for PJS, numerous important findings concerning the LKB1-AMPK pathway have been reported, increasing our understanding of its physiological and pathological roles in cell polarity. However, there are some issues about the establishment of cell polarity that remain unclear: What controls the selectivity of LKB1 for each of the 14 AMPK-related protein kinases, and how does LKB1 regulate these kinases? How does AMPK control the phosphorylation level of each of its substrates and exert different effects on the maintenance of metabolic homeostasis and the establishment of cell polarity? The activity of LKB1 might be continuously maintained at a certain level, as its *in vivo* activity has been reported to undergo little change, suggesting that the activity of AMPK-related protein kinases would also be maintained at a specific level. Thus, AMPK may be able to maintain fundamental cell functions, including cell polarity by LKB1, and regulate many additional functions, depending on the energy status of the cell, by controlling the phosphorylation levels of its substrates. Our results from a screen for AMPK substrates showed that CLIP-170 was the most susceptible substrate for phosphorylation by AMPK. From this finding, it is reasonable to assume that CLIP-170 is one of the main regulators of cell polarity, as CLIP-170 appears to be phosphorylated at a lower level than the known substrates. Further investigations will elucidate these issues more clearly and contribute to the advancement of the field of cell biology.
